# Hypoxia Alters Epigenetic and *N*-Glycosylation Profiles of Ovarian and Breast Cancer Cell Lines *in-vitro*

**DOI:** 10.3389/fonc.2020.01218

**Published:** 2020-07-29

**Authors:** Gordon Greville, Esther Llop, Chengnan Huang, Jack Creagh-Flynn, Stephanie Pfister, Roisin O'Flaherty, Stephen F. Madden, Rosa Peracaula, Pauline M. Rudd, Amanda McCann, Radka Saldova

**Affiliations:** ^1^GlycoScience Group, The National Institute for Bioprocessing Research and Training (NIBRT), Dublin, Ireland; ^2^UCD School of Medicine, College of Health and Agricultural Science (CHAS), University College Dublin (UCD), Dublin, Ireland; ^3^Biochemistry and Molecular Biology Unit, Department of Biology, University of Girona, Girona, Spain; ^4^Biochemistry of Cancer Group, Girona Biomedical Research Institute (IDIBGI), Girona, Spain; ^5^Data Science Centre, Division of Population Health Sciences, Royal College of Surgeons in Ireland (RCSI), Dublin, Ireland; ^6^Analytics Group, Bioprocessing Technology Institute, Astar, Singapore; ^7^UCD Conway Institute of Biomolecular and Biomedical Research, University College Dublin (UCD), Dublin, Ireland

**Keywords:** hypoxia, ovarian cancer, breast cancer, glycosylation, epigenetics

## Abstract

**Background:** Glycosylation is one of the most fundamental post-translational modifications. Importantly, glycosylation is altered in many cancers. These alterations have been proven to impact on tumor progression and to promote tumor cell survival. From the literature, it is known that there is a clear link between chemoresistance and hypoxia, hypoxia and epigenetics and more recently glycosylation and epigenetics.

**Methods and Results:** Our objective was to investigate these differential parameters, in an *in vitro* model of ovarian and breast cancer. Ovarian (A2780, A2780cis, PEO1, PEO4) and triple negative breast cancer (TNBC) (MDA-MB-231 and MDA-MB-436) cells were exposed to differential hypoxic conditions (0.5–2% O_2_) and compared to normoxia (21% O_2_). Results demonstrated that in hypoxic conditions some significant changes in glycosylation on the secreted *N*-glycans from the ovarian and breast cancer cell lines were observed. These included, alterations in oligomannosylated, bisected glycans, glycans with polylactosamine extensions, in branching, galactosylation and sialylation in all cell lines except for PEO1. In general, hypoxia exposed ovarian and TNBC cells also displayed increased epithelial to mesenchymal transition (EMT) and migration, with a greater effect seen in the 0.5% hypoxia exposed samples compared to 1 and 2% hypoxia (*p* ≤ 0.05). SiRNA transient knock down of *GATA2/3* transcription factors resulted in a decrease in the expression of glycosyltransferases *ST3GAL4* and *MGAT5*, which are responsible for sialylation and branching, respectively.

**Conclusions:** These glycan changes are known to be integral to cancer cell survival and metastases, suggesting a possible mechanism of action, linking GATA2 and 3, and invasiveness of both ovarian and TNBC cells *in vitro*.

## Introduction

Ovarian cancer and breast cancer, although organ specific neoplasms, have genomic and biological similarities. For example, **(i)** ~5% of breast cancers [mostly triple negative breast cancers (TNBCs) (1)], and 10% of ovarian cancers (2), carry a BRCA1/2 mutation, **(ii)** both are associated with high relapse rates and high proportions of chemo-resistance and **(iii)**, both display an innately hypoxic tumor microenvironment which has been shown to up regulate a “*stem cell-like*” property; a property integral to the progression of these tumors (3–5). As the tumor grows in size, so does its energy demand and its need for oxygen. When the oxygen demand outweighs the availability of oxygen in the tumor it becomes hypoxic. The association between hypoxia and chemo-resistance has been widely reported (5). In addition, Watson et al. (6) have demonstrated epigenetic signatures which promoted and maintained a hypoxic-adapted cellular phenotype in benign prostate epithelial cells, with a potential role in prostate cancer development (6). Shahrzad et al. (7) published that the hypoxic conditions observed in tumors resulted in the global DNA hypomethylation we traditionally associate with cancer (7). Methionine adenosyltransferase (MAT) is encoded by two genes in mammals, namely MAT1A and MAT2A, and catalyzes the production of S-adenosyl methionine (SAM) (8). SAM is a methyl donor substrate, used in many cellular processes, including DNA and histone methylation (9). Liu et al. (9) have shown that *MAT2A* has a hypoxia responsive element (HRE) motif within its promoter region, where HIF-1α binds, resulting in the up regulation of *MAT2A* gene transcription in hypoxia (1%) (9). GATA3, a transcription factor (TF) involved in the development of numerous biological responses and specific tissue development, has also been shown to interact with, and stabilize HIF-1α (10). It has also been demonstrated that the GATA TF family are heavily dependent on CpG island promoter methylation for activation/repression (11). Interestingly, *GATA3* a gene, which has a role in Th2-cell differentiation, shows differential methylation status on Th1 cells compared toTh2 cells (12).

The potential link between hypoxia and glycosylation is less well-understood. MiR-200b, known to be downregulated in hypoxia (1%), is associated with fucosylation (13, 14). Shirato et al. (15) have also shown that 1% hypoxia alters glucose metabolic fluxes, that can modulate cellular glycosylation patterns (15). In addition, Ren et al. (16) have demonstrated that certain *N*-linked glycosylation sites on integrin-α3 are important for cell to cell adhesion, and that modification to these glycans results in a potential EMT phenotype (16). DNA methylation and glycosylation have been shown to be involved in the regulation of EMT, apoptosis, senescence, and autophagy (17–24). It is therefore likely, that the tumor microenvironment of hypoxia will affect these processes. It is also accepted that activation of HIF signaling, influences numerous steps in the metastatic cascade, including invasion, migration, extravasation and intravasation (25).

In this study, our objective was to demonstrate the impact of hypoxia on glycosylation profiles from the secreted glycoproteins of ovarian and breast cancer cells *in-vitro*, and determine the epigenetic alterations that may underlie these altered glycosylation profiles.

## Materials and Methods

### Tissue Culture

A2780, PEO1, PEO4, MDA-MB-231, MDA-MB-436 were authenticated in 2015/16 by DDC medical, via short tandem repeat (STR) profiling. A2780cis cells were obtained from The European Collection of Authenticated Cell Cultures (ECACC). All cell lines were maintained in RPMI 1640 medium supplemented with 10% v/v fetal calf serum (FCS) and 2 mM L-glutamine. PEO1 and PEO4 were further supplemented with 1 mM sodium pyruvate. All cells were cultured at 37°C with 5% CO_2_. A2780cis cells were treated with 1 μM cisplatin every 2/3 passages, to ensure chemo-resistance was maintained. All cell lines were also routinely tested for *Mycoplasma* contamination in the UCD Conway Institute of Biomolecular and Biomedical Research UCD, Dublin.

### Incubation of Cells in Hypoxic (0.5, 1, and 2%) Conditions

To ensure a sustainable deficiency of oxygen, cells were incubated in a hypoxic chamber under 0.5, 1, and 2% O_2_ for a 24 and 48 h period, respectively, with media preconditioned to the relative oxygen environment for at least 12 h prior to culturing. The 48 h hypoxia exposed cell line media was replenished with preconditioned media after 24 h. Following incubation, the cells were immediately harvested and depending on the analysis being performed, cell pellets were fixed in 70% methanol, or frozen at −20°C, for up to 6 weeks or −80°C for longer periods for western blot and subsequent TaqMan® RT-qPCR analyses.

### Flow Cytometry

Cells were harvested by trypsinisation, fixed in 70% methanol and subsequently stained with Propidium Iodide (PI) (Sigma) and an anti-5′methylCytidine (5′MeC) (Eurogentec) primary antibody. Prior to staining with 5′MeC, the cells were pre-incubated with 1 M HCl at 37°C for 1 h. IgG negative controls were used at the same concentration as the primary antibody. Secondary antibody staining was conducted using an FITC conjugated rabbit anti-mouse secondary antibody (Dako). Analyses were performed on an Accuri C6 flow cytometer and results assessed using FCS Express software (De Novo).

### Cell Secretome Harvesting

Supernatants were spun down and concentrated using Amicon Ultra-15 10K ultrafiltration (Millipore), to a final volume of below 200 μL. Proteins were precipitated with a half volume of 50:50 TCA: acetone (w/v) on ice. The mixture was then incubated for 45 min on ice and centrifuged at 13,000 rpm for 5 min. The resultant pellet was washed with cold acetone and centrifuged again at 13,000 rpm for 5 min. This final pellet was dried and resuspended in sample buffer (2% SDS, 62.5 mM TRIS pH 6.6) for subsequent *N*-glycan analysis.

### Glycan Analysis

*N-*glycans were released from glycoproteins in samples, by *in situ* digestion with Peptide *N-*glycosidase F (PNGase F; Prozyme) in-gel blocks (26). Briefly, samples from cell secreted glycoproteins, were reduced and alkylated, and subsequently set into SDS-gel blocks. *N-*glycans were released by adding 50 μL of 1 U/400 μL PNGase F in 20 mM NaHCO_3_, pH 7.2. The reduced terminus of the oligosaccharides were then fluorescently labeled with 2-aminobenzamide (2AB) by reductive amination (27). The samples were then run on HILIC-UPLC.

### Hydrophilic Interaction Liquid Chromatography-Ultra Performance Liquid Chromatography (HILIC-UPLC)

HILIC-UPLC was carried out on a BEH Glycan 1.7 μM 2.1 × 150 mm column (Waters), on an Acquity UPLC H-Class (Waters), coupled with an Acquity fluorescence detector. Solvent A was 50 mM formic acid adjusted to pH 4.4 with ammonia solution. Solvent B was acetonitrile. The column temperature was set to 40°C. The following conditions were used; 30 min method using a linear gradient of 30–47% A at 0.56 mL/min in 23 min (28). Samples of 10 μL volumes, were injected in 70% acetonitrile. Fluorescence was measured at 420 nm, with excitation at 330 nm. The system was calibrated using a 2AB-labeled glucose oligomers, to create a dextran ladder with retention times of all identified peaks expressed as glucose units (GUs) (29).

### Ultra-Performance Liquid Chromatography-Fluorescence-Mass Spectrometry (UPLC-FLR-MS)

Lyophilised cell line secretome samples, were reconstituted in 3 μl of water and 9 μl acetonitrile. Online coupled fluorescence (FLR)-mass spectrometry detection was performed using a Waters Xevo G2 QTof with Acquity® UPLC (Waters Corporation, Milford, MA, USA) and BEH Glycan column (1.0 × 150 mm, 1.7 μm particle size). For MS acquisition data, the instrument was operated in *negative-sensitivity* mode with a capillary voltage of 1.80 kV. The ion source block and nitrogen desolvation gas temperatures were set at 120 and 400°C, respectively. The desolvation gas was set to a flow rate of 600 L/h. The cone voltage was maintained at 50V. Fullscan data for glycans were acquired over m/z range of 450–2,500. Data collection and processing were controlled by MassLynx 4.1 software (Waters Corporation, Milford, MA, USA). The fluorescence detector settings were as follows ex = 330 nm, em = 420 nm. Sample injection volume was 8 μL. The flow rate was 0.150 mL/min and column temperature was maintained at 60°C; solvent A was 50 mM ammonium formate in water (pH 4.4) and solvent B was acetonitrile. A 40 min linear gradient was used, and was as follows: 28% (v/v) A for 1 min, 28–43% (v/v) A for 30 min, 43–70% (v/v) A for 1 min, 70% (v/v) A for 3 min, 70–28% (v/v) solvent A for 1 min and finally 28% (v/v) A for 4 min.

### Exoglycosidase Digestions

The 2AB-labeled oligosaccharides were digested in a volume of 10 μL for 18 h at 37°C in 50 mM sodium acetate buffer, pH 5.5 (with the exception of jack bean α-mannosidase (JBM), where the buffer was 100 mM sodium acetate, 2 mM Zn^2+^, pH 5.0), using arrays of the following enzymes: *Arthrobacter ureafaciens* sialidase (ABS), 0.5 U/mL; *Streptococcus pneumoniae* sialidase (NAN1), 1 U/mL; bovine testes β-galactosidase (BTG), 1 U/mL; *Streptococcus pneumoniae* β-galactosidase (SPG), 0.4 U/mL; bovine kidney α-fucosidase (BKF), 1 U/mL; β-*N*-acetylglucosaminidase cloned from *Streptococcus pneumonia*, expressed in *E. coli* (GUH), 8 U/mL (Prozyme) or 400 U/mL (NEB); jack bean α-mannosidase (JBM), 60 U/mL; almond meal α-fucosidase (AMF), 0.4 mU/mL. All enzymes were purchased from Prozyme and GUH, was also purchased from NEB. Following digestion, the enzymes were removed by filtration through a Pall 10 kDa MWCO microcentrifuge filtration device (Pall cat no. 516-8491), and the oligosaccharides were analyzed by HILIC-UPLC.

### Feature Analysis

Glycan peaks were pooled based on similar structural or compositional features of the peak glycan members. Features pertaining to a peak were determined based on the major glycan members of that peak. (See Table S1).

### Electrophoresis and Western Blot Analysis

Whole cell extracts were separated by SDS-PAGE using 4–15% precast TGX gels (BioRad), and transferred onto PVDF membranes using the Trans-Blot® Turbo™ system (Biorad). Blots were blocked and incubated with rabbit monoclonal antibodies targeting **PARP** (1:1000, Cell Signaling Technologies), **p21** (1:1000, Abcam), **p16** (1:1000, Abcam), **N-Cadherin** (1:1000, Abcam), **GATA2** and **GATA3** (Sigma, 1:1000); rabbit polyclonal antibody targeting **LC3** (1:1000, Abcam); and mouse monoclonal antibodies targeting **E-Cadherin** (1:500, Abcam), **Rb** (1:1000, Abcam), **ST3GAL4** (Abcam, 1:500) and **MGAT5** (Abcam, 1:400). The membranes were then incubated with a secondary goat anti-mouse or anti-rabbit antibody (1:5000, Abcam). The blots were developed using TMB for enzymatic colourimetric detection. To analyse protein loading, the mouse monoclonal, **α-Tubulin** antibody (1:10,000; Santa Cruz, CA, USA) was used. Western blots were quantified using ImageJ^TM^ software (FIJI).

### RT-qPCR (Reverse Transcription Quantitative PCR)

Eight gene transcripts were analyzed comprising of **(i)** glycosyltransferases mannosyl-(α1,6-)-glycoprotein β1,6-N-acetyl-glucosaminyltransferase **(*MGAT5*)** and β-galactoside α2,3-sialyltransferase 4 **(*ST3GAL4*), (ii)** enzymes in the sugar nucleotide donor pathway GDP-mannose-4,6-dehydratase **(*GMDS*)**, mannose phosphate isomerase **(*MPI*)** and tissue specific transplantation antigen P35B **(*TSTA3*)** and **(iii)** TFs, ***GATA1***, ***GATA2*** and ***GATA3*** identified in a preliminary *in-silico* study (Dr. Stephen Madden, DCU). Firstly, total RNA was isolated from the cells using an RNeasy Mini kit (Qiagen, Cat No. 74104). The RNA (500 ng) was subsequently reverse transcribed using the High-Capacity cDNA Reverse Transcription Kit (Applied Biosystems). RT-qPCR was then performed using the following TaqMan assay probes ***ST3GAL4*:** Hs00920871 m1, ***MGAT5:*** Hs00159136 m1, ***TSTA3*:** Hs00163023 m1, ***GMDS*:** Hs00155276 m1, ***MPI:*** Hs00159228 m1, ***GATA1*:** Hs01085823 m1, ***GATA2:*** Hs00231119 m1, ***GATA3*:** Hs00231122 m1 (Thermo Fisher), on a 7500 Fast Real-Time PCR System (Applied Biosystems) to analyse the expression of each RNA by the comparative delta Ct method, normalized using the TATA box binding protein (TBP): Hs99999910 m1. TATA is a constitutive gene and does not vary in the treatments (30–32). Three technical replicates were performed for each sample and each gene and PCR assays were carried out in duplicate. Results were expressed as mean ± SD values.

### Migration Assay

The Oris™ Cell Migration Assay (Platypus Technologies) was used to assess cell migration in all cell lines under normoxia and hypoxia conditions. Conditions were optimized based on the manufacturer's instructions. Briefly, 2.5 × 10^4^-5 × 10^4^ cells/well/100 μL were added into stopper-loaded wells and incubated overnight to permit cell attachment. The stoppers were removed from the wells to allow cell migration and the wells were gently washed with PBS. Complete cell culture medium was then added and the cells/wells incubated for 48 h. Each well was then washed with sterile PBS to remove any dead cells. The remaining cells were then fixed using 70% methanol diluted in water and incubated in Coomassie Instant Blue for 20 min. A reading of each well was taken on a microplate reader (Perkin Elmer) at 595 nm. As a negative control, a set of stoppers were left in place until the experiment was completed, so the cells were contained within the stoppers and no migration occurred in this sample.

### Transient GATA Knockdown

Cells were transiently transfected with 100 nM of either siRNA targeting GATA2 (A2780, A2780cis) or GATA3 (PEO1, PEO4) (Dharmacon) transcripts, and 25nM of scrambled control siRNA using DharmaFECT1 (Dharmacon) transfection reagent. DharmaFECT1 volumes and siRNA concentrations were optimized for a 12 well-plate according to the manufacturer's protocol (Figure S1). Briefly, cells were seeded at an optimal density, so that at the time of transfection the cells were 60–70% confluent. For A2780 and A2780cis this was 4 × 10^5^ cells/well the day before transfection and for PEO1 and PEO4 cells this was 6 × 10^5^ cells/well. On the day of transfection, separate tubes were prepared with the appropriate volume of DharmaFECT1 (2 μL/well), scrambled siRNA (25 nM final concentration) and targeted siRNA (100 nM final concentration), diluted in serum free RPMI media. The DharmaFECT1 was then mixed with the siRNA and allowed to stand at room temperature for 20 min. At the same time, the media was removed from the 12 well-plates, and replaced with 800 μL/well of fresh complete growth media. 200 μL of DharmaFECT1/siRNA mix was then added to the appropriate wells. The cells were incubated at 37°C for 24 and 48 h.

### Statistical Analysis

All data are expressed as the means ± the standard deviation (SD). Statistical analyses were performed using SPSS statistical software for Windows (version 24.0; SPSS Inc.). For significances in glycan data, individual glycan peaks and features, the HILIC-UPLC data were logit transformed, and examined using a MANOVA with *post-hoc* analysis using Tukey's Honest Significant difference. For other data, such as the RT-qPCR, Western blot densitometry and migration assays, *t*-tests, one- or two-way ANOVAs were used as was deemed appropriate. A *p* < 0.05 was considered statistically significant.

### Heatmap Histograms

Histograms indicating fold changes in hypoxia conditions in comparison to normoxia in Figures were created using Hierarchial Clustering Explorer HCE 3.5 software.

## Results

### Hypoxia (0.5–2% O_2_) Alters the Global Methylation of Breast and Ovarian Cancer Cell Lines *in-vitro*

**Figure 1:**
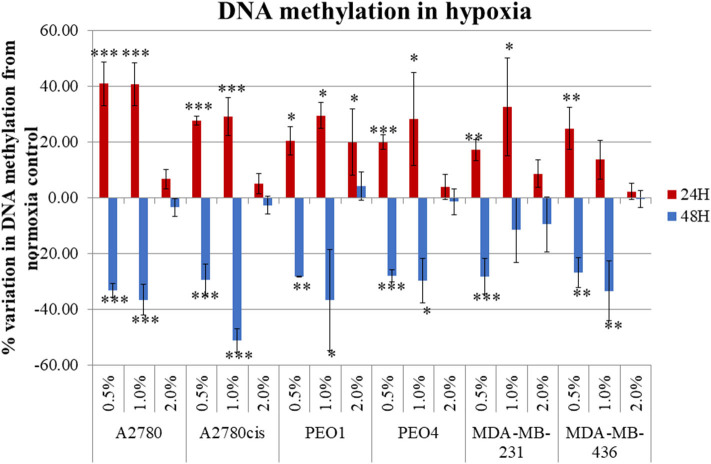
Hypoxia alters global DNA methylation. The histogram represents the average global DNA methylation variation compared to a normoxic control for the 6 cell lines analyzed. Each cell line was exposed to 0.5, 1.0, and 2.0% for 24 h (blue) and 48 h (red). The error bars were calculated from the SD of 3 biological replicates. Significant changes are starred: * *P* ≤ 0.05; **P ≤ 0.01; ***P ≤ 0.001 (one-way ANOVA).

Four ovarian cancer (A2780, A2780cis, PEO1, and PEO4) and two TNBC (MDA-MB-231 and MDA-MB-436) cell lines were selected for this study. A2780/A2780cis and PEO1/PEO4 are pairs of chemosensitive/chemoresistant ovarian cancer cell lines and were selected to determine if our observed results differ between chemosensitive/chemoresistant cancer cell lines. The inclusion of two TNBC cell lines, allowed for comparison with another tumor type, which like ovarian cancer, is very prone to a hypoxic tumor microenvironment *in-vivo*. All cells were cultured in 0.5, 1, and 2% O_2_ percentages and compared to normoxic conditions (21% O_2_) for 24 and 48 h, to determine the impact if any that hypoxia had on global cellular DNA methylation. Hypoxic exposure (0.5 and 1% O_2_ levels), significantly altered the global DNA methylation profile compared with the cells cultured under normoxic conditions (21% O_2_) (*p* ≤ 0.05). There was a significant increase in DNA methylation (*p* ≤ 0.05) (up to 60%) after 24 h and significant decrease in DNA methylation (*p* ≤ 0.05) (up to 60%) after 48 h incubation under these hypoxic conditions for all cell lines (Figure 1).

### *N*-Glycosylation Is Dysregulated in Hypoxia Exposed Cells

**Figure 2:**
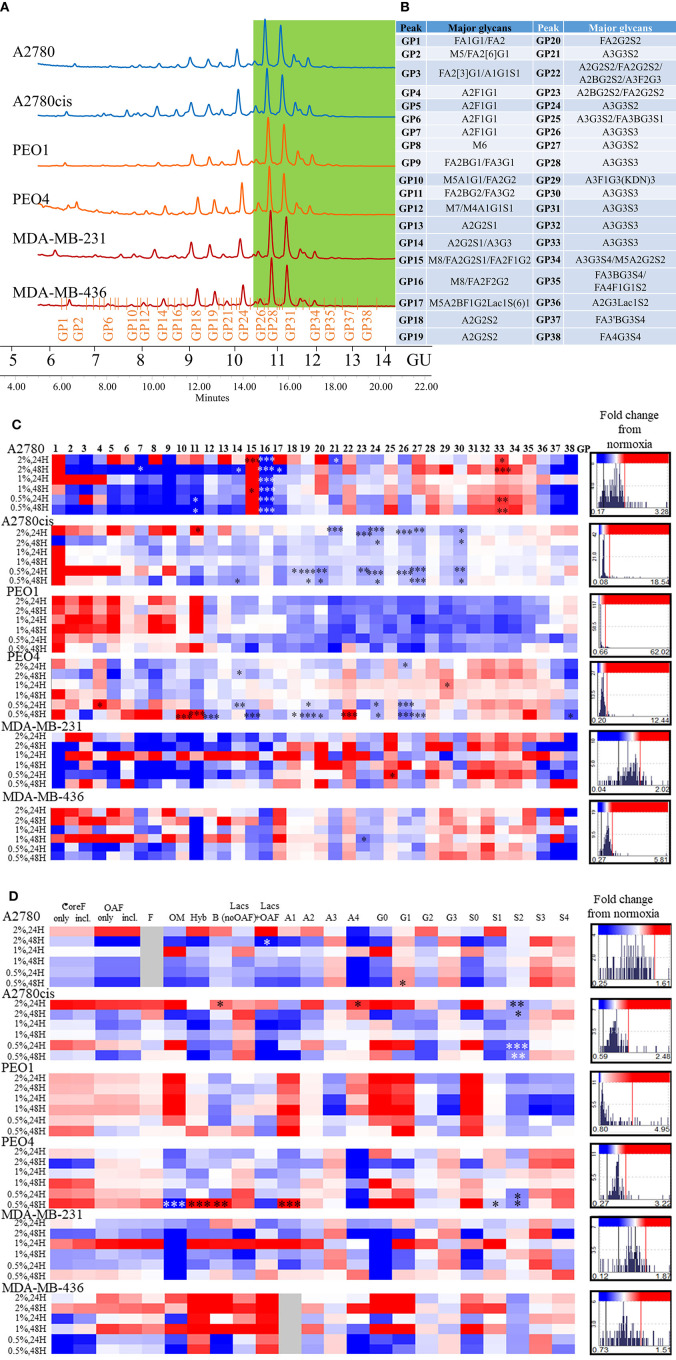
Glycosylation in hypoxia. **(A)** Typical UPLC chromatograms of secreted *N*-glycome separated into 38 peaks from ovarian chemosensitive (in blue), chemoresistant (in orange) and triple negative breast cancer (TNBC) (in red) cells. Highly branched and sialylated glycans are highlighted in green. **(B)** Main glycans in each peak. Structure abbreviations: All *N*-glycans have two core GlcNAcs; F at the start of the abbreviation indicates a core-fucose α1,6-linked to the inner GlcNAc; Mx, number of mannose on core GlcNAcs; Ax, number of antenna (GlcNAc) on trimannosyl core; A2, biantennary with both GlcNAcs as β1,2-linked; A3, triantennary with a GlcNAc linked β1,2 to both mannose and the third GlcNAc linked β1,4 to the α1,3 linked mannose; A3', isomer with the third GlcNAc linked β1-6 to the α1-6 linked mannose; B, bisecting GlcNAc linked β1,4 to β1,3 mannose; Gx, number of β1,4 linked galactose on antenna; Fx, number of fucose linked α1,3 to antenna GlcNAc; Sx, number of sialic acids linked to galactose; **(C)** Plotted peak areas of the *N*-glycans from secreted glycoproteins from ovarian and breast cancer cell lines. **(D)** Plotted features calculated from the peak areas of the *N*-glycans from secreted glycoproteins from ovarian and breast cancer cell lines [fold difference between hypoxia conditions and control normoxia (21% O_2_)]. Significant changes are starred: *P ≤ 0.05; **P ≤ 0.01; ***P ≤ 0.001 (MANOVA). The histograms on the right side of the **(C,D)** are indicating increasing (in red) and decreasing (in blue) trends in GPs in hypoxia conditions comparing to normoxia.

All differential hypoxia cultures (0.5–2% O_2_) and normoxia controls (21% O_2_), at the 24and 48 h timepoint, were analyzed for detailed *N-*glycan composition from secreted glycoproteins. Profiles from each cell type are represented in Figure 2A. Structural assignments were made using the Glycostore database (https://glycostore.org/) (33), exoglycosidase digestion arrays as well as mass spectrometry (MS). The main glycans are presented in Figure 2B and detailed assignments using UPLC and MS presented in Tables S2, S3, respectively. Table S1 outlines all glycans in all cell lines and how the individual glycan peaks were pooled into the common features. Table 1 summarizes the glycomic changes in all cell lines. Figure 2C shows colored fold changes in the percentage areas in the individual peaks from all samples in hypoxia conditions compared to normoxia controls, increases are in red and decreases in blue color. Those stared, represent significant changes (*p* ≤ 0.05).

**Table 1:** Summary of glycomic changes in all cell lines.

**Significantly altered glycans in hypoxia**	**A2780**	**A2780cis**	**PEO1**	**PEO4**	**MB-231**	**MB-436**
A2F1G1						
M5A1G1						
FA2BG2, FA3G2						
M7						
A2G2S1, A3G3						
M8, FA2F2G2						
M8						
M5A2BF1G2Lac1S(6)1						
A2G2S2						
A2G2S2*-isomer of higher GU value						
FA2G2S2						
A3G3S2						
A3G3S3						
A3F1G3KDN3						
FA3BG3S4						
**Glycan features**						
Oligomannose						
Hybrids						
Bisecting glycans (B)						
Lacs with outer arm fucose						
Monoantennary glycans (A1)						
Tetraantennary glycans (A4)						
Monogalactosylated glycans (G1)						
Monosialylated glycans (S1)						
Disialylated glycans (S2)						

*Increased glycans are in red and decreased in green*.

*Any changes seen in hypoxia are summarized and highlighted (from 0.5 to 2%)*.

***Structure abbreviations:** All N-glycans have two core GlcNAcs; F at the start of the abbreviation indicates a core-fucose α1,6-linked to the inner GlcNAc; Mx, number (x) of mannose on core GlcNAcs; Ax, number of antenna (GlcNAc) on trimannosyl core; A2, biantennary with both GlcNAcs as β1,2-linked; A3, triantennary with a GlcNAc linked β1,2 to both mannose and the third GlcNAc linked β1,4 to the α1,3 linked mannose; A4, GlcNAcs linked as A3 with additional GlcNAc β1,6 linked to α1,6 mannose; B, bisecting GlcNAc linked β1,4 to β1,3 mannose; Gx, number (x) of β1,4 linked galactose on antenna; F(x), number (x) of fucose linked α1,3 to antenna GlcNAc; Sx, number (x) of sialic acids linked to galactose. Where not specified, the glycan contains sialic acid linked α2,3 and α2,6*.

In general, peaks were identified as being significantly increased or decreased (*p* ≤ 0.05), 0.08–62.02-fold, in all cell lines. To see the specific type of glycans affected, the glycans were pooled into common features (Figure 2D). Significant alterations are in oligomannosylated, bisected glycans, glycans with polylactosamine extensions, in branching, galactosylation and sialylation (*p* ≤ 0.05) (Figure 2D, Table 1). Generally, there is a trend for a decrease in core fucosylated and oligomannosylated glycans, and an increase in outer arm fucosylation, polylactosamine extensions and sialylation on all cell lines (changes range from 0.12 to 4.95-fold) (Figure 2). The most significant of these glycosylation changes described, were identified on A2780 and A2780cis cell lines (*p* ≤ 0.05). The results for PEO1 were different from all other cell lines, but were not statistically significant (Figure 2).

The presence of ketodeoxynonulosonic acid (KDN, a form of sialic acid), on triantennary glycans, containing core and outer arm fucose, was found (3.8–9% of the total glycans, present as a major glycan in GP29 in all cell lines) (Table S2). This peak was significantly increased in PEO4 at 1% hypoxia for 24 h (*p* ≤ 0.05) (Figure 2C).

After the effect of hypoxia on DNA methylation and glycosylation of secreted glycoproteins was evaluated, additional cancer cell hallmarks were examined in relation to potential metastatic capability under hypoxic conditions, which could be related to the observed glycomics changes. These features were the epithelial to mesenchymal transition (EMT) and cellular migration. Cellular apoptosis, senescence and autophagy were also examined.

### Hypoxia Increases EMT and Migration in All Cancer Cell Lines *in vitro*

**Figure 3:**
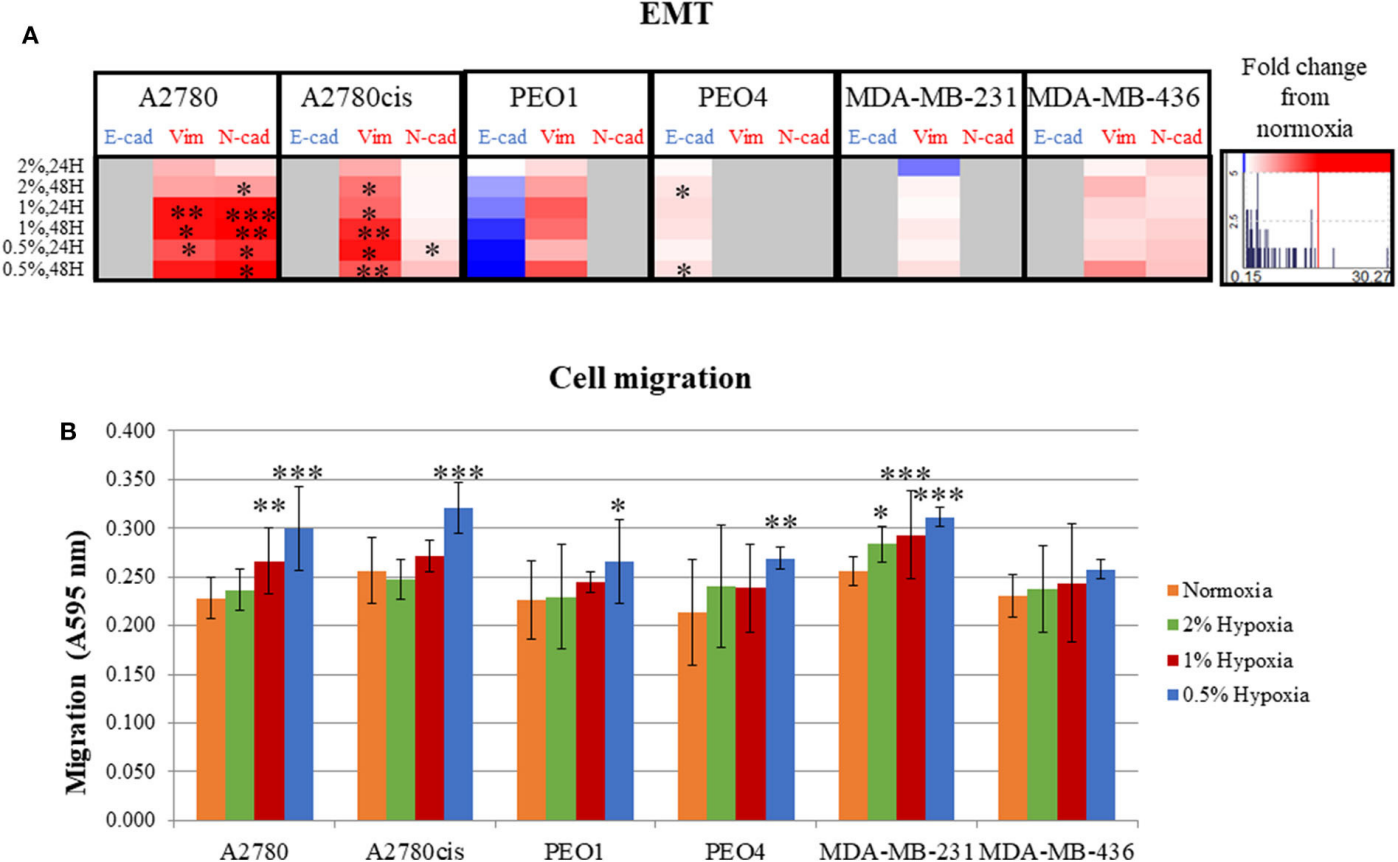
Hypoxic exposure increases migration of breast and ovarian cancer cells; association with markers of EMT. **(A)** Fold change of the protein expression of E-cadherin, vimentin and N-cadherin as determined by densitometry (Image J)(Western blots are shown in Figure S3). Each condition was carried out on three biological replicates (*n* = 3). **(B)** Quantification of absorbance readings from the Oris migration assay following hypoxic exposure. Cells were stained with Coomassie Instant Blue and the absorbance was read at 595 nm. Error bars were calculated from 3 to 6 independent experiments with 4 technical replicates per experiment. Significant changes are starred: *P ≤ 0.05; **P ≤ 0.01; ***P ≤ 0.001 (*T*-test and ANOVA). The histograms on the right side of the figure are indicating increasing (in red) and decreasing (in blue) trends in EMT markers in hypoxia conditions comparing to normoxia.

Following determination of the glycosylation changes with exposure to hypoxia (0.5–2% O_2_) compared to normoxia cultured cells, migration and EMT were assessed to determine if there was a link between altered glycan structures and changes in migratory capability. Firstly, markers related to EMT were analyzed in all cell lines. Specifically, E-cadherin, which is downregulated in EMT and *N*-cadherin and vimentin, both known to be upregulated in EMT (34) (Figure 3A, Figure S2). EMT was significantly increased in A2780 and A2780cis cell lines (*p* ≤ 0.05) (up to 30.27 times), with an increased trend observed in all other cell lines with the exception of PEO4. Specifically for PEO4, E-cadherin expression was seen to increase (^*^), indicating a decrease in EMT. The two TNBC cell lines MDA-MB-231 and MDA-MB-436, demonstrated variability in E-cadherin protein expression following hypoxia exposure. E-cadherin protein expression was not detectable in A2780, A2780cis, MDA-MB-231 and MDA-MB-436. N-cadherin expression was not detected in PEO1, PEO4 and MDA-MB-231. Vimentin was not detected in PEO4.

The Oris™ Cell Migration Assay was used to assess changes in migration potential (Figure 3B) following 48 h exposure to 0.5% hypoxia. Results show that 0.5% hypoxic exposure significantly increased migration in all cells (*p* ≤ 0.05) except for MDA-MB-436. A 1% hypoxia exposure for 48 h, also increased migration in A2780 and MDA-MB-231 (*p* ≤ 0.005) and 2% for 48 h in MDA-MB-231 cells (*p* ≤ 0.05).

Looking at both the EMT and migration in hypoxia conditions (0.5–2% O_2_), the ovarian PEO1 and TNBC MDA-MB-436 cell lines were the least affected by hypoxia (Figures 2C,D).

Overall, the increased time of exposure (48 h) and lower O_2_ percentages applied (0.5%) resulted in more significant increases observed in their EMT and migration potential (*p* ≤ 0.05) (Figures 2C,D).

### Senescence and Autophagy Are Specifically Altered in Hypoxia, Apoptosis Increases Only in Chemosensitive Cells

**Figure 4:**
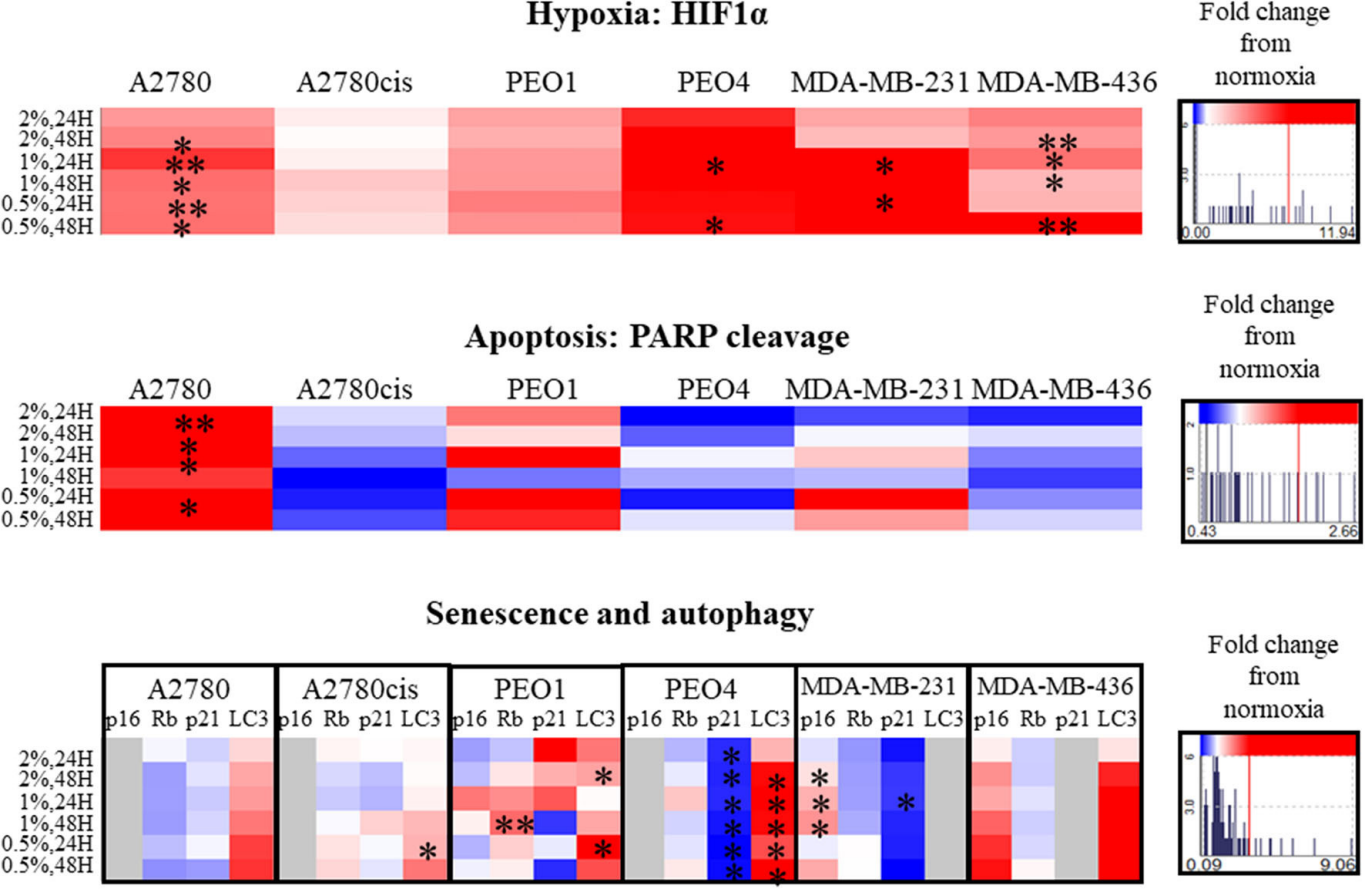
Hypoxia alters apoptosis, senescence and autophagy specifically. Protein expression of HIF1α, PARP, p16, p21, Rb, and LC3 as determined by densitometry (Image J)(Western blots are shown in Figure S4). Each condition was carried out on three biological replicates (*n* = 3). Significant changes are starred: *P ≤ 0.05; **P ≤ 0.01 (*T*-test). The histograms on the right side of the figure are indicating increasing (in red) and decreasing (in blue) trends in hypoxia, apoptosis, senescence and autophagy markers in hypoxia conditions comparing to normoxia.

Hypoxia induction was confirmed by the increased protein expression of HIF1α in all conditions, significantly in A2780, PEO4, MDA-MB-231 and MDA-MB-436 cell lines (*p* ≤ 0.05, up to 11.94 times) (Figure 4). There were no significant cell cycle alterations in hypoxia (Figure S3).

PARP cleavage was significantly increased only in A2780 in hypoxia conditions (0.5–2% O_2_, *p* ≤ 0.05, up to 2.66 times) compared to A2780 cis. Although not significant, PARP cleavage increased in the chemosensitive PEO1 and decreased in the chemoresistant (PEO4) ovarian cells. For the 2 TNBC cell lines, although not reaching significance, there was increased PARP cleavage in MDA-MB-231 with increasing hypoxia, with no PARP cleavage evident in the MDA-MB-436 cells, these changes were from 0.43 up to 2.66 times (Figure 4).

The senescence marker p21 was significantly decreased in all hypoxic exposure in PEO4 cells with a concomitant increase in the autophagy marker LC3. The senescence markers p16, Rb, and p21 were variably expressed in the cell lines in hypoxia (0.5–2% O_2_) (Figure 4). For the TNBC cell lines, there was a significant increase in P16 for the MDA-MB-231, *p* ≤ 0.05. However, p16 in hypoxia was not expressed in A2780, A2780cis and PEO4. P21 decreased in PEO4 and MDA-MB-231 (*p* ≤ 0.05), and it was not expressed in MDA-MB-436. The autophagy marker LC3 increased in all cell lines (significantly in A2780cis, PEO1 and PEO4, *p* ≤ 0.05). However, the TNBC cell line MDA- MDA-MB-231, did not express LC3 under these conditions.

### Hypoxia Alters the Expression Levels of *MGAT5* and *ST3GAL4* Glycosyltransferase Genes Which Correlate With the Expression of Transcription Factors (TFs) *GATA2* and *GATA3* in Ovarian Cancer Cell Lines

**Figure 5:**
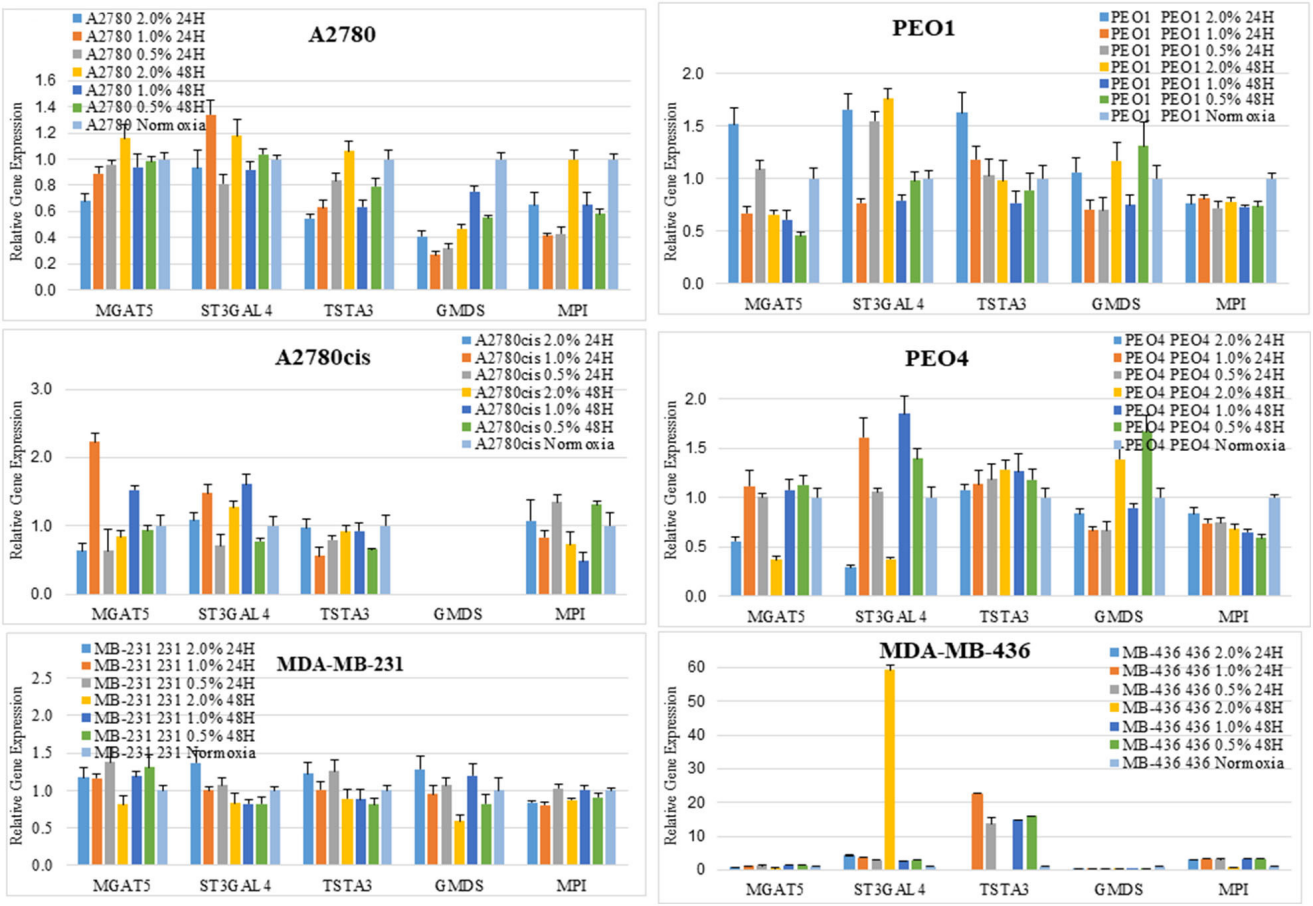
Changes seen in UPLC *N-*glycan profiles, are associated with the mRNA expression levels of glycosyltransferases. Quantitative RT-PCR gene expression analyses were undertaken for selected glycosyltransferases (*MGAT5* and *ST3GAL4*) and enzymes involved in the sugar nucleotide donor pathway (*MPI, TSTA3*, and *GMDS*) following the culturing of cells in differential hypoxic conditions (0.5–2% O_2_) compared to normoxia (21% O_2_) controls. The relative expression level of each gene was calculated according to the ddCt normalized with TATA-Box Binding Protein (TBP). Each condition was undertaken on one biological replicate (*n* = 1) and three technical replicates (*n* = 3).

**Figure 6:**
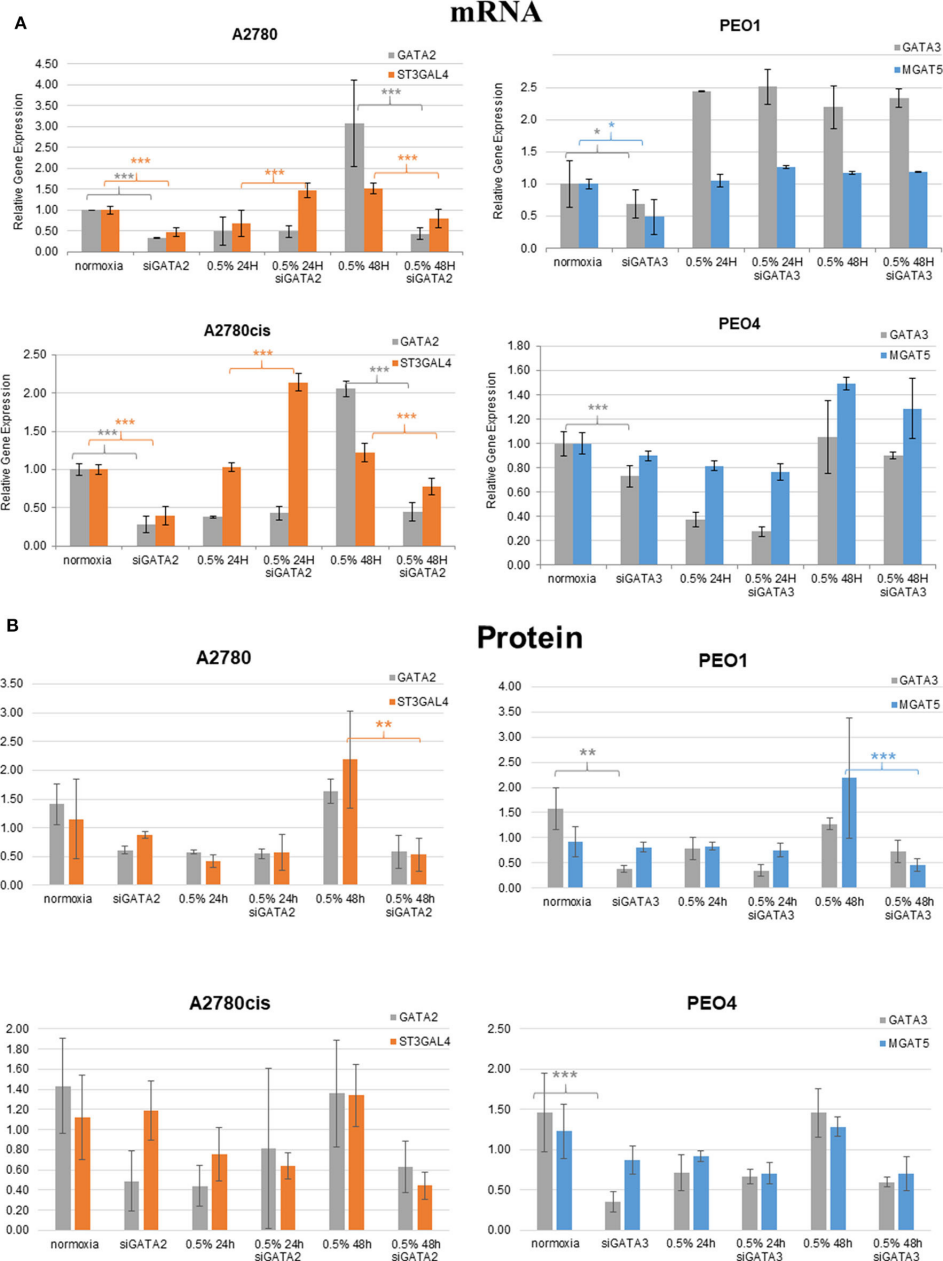
Hypoxia alters mRNA expression of MGAT5 and ST3GAL4 through the regulation of GATA2 and 3. Expression of the GATA transcription factors (GATA2 and 3) and the glycosyltransferases MGAT5, ST3GAL4, in normoxia (21% O_2_) and 0.5% hypoxia conditions, before and after siGATA knockdown at **(A)** the gene expression level, measured by quantitative RT-PCR analysis. Each sample represents of 2 biological replicates (*n* = 2). The relative expression level of each gene was calculated according to the ddCt normalized with TBP; and at **(B)** the protein level as determined by densitometry (Image J)(Western blots are in Figure S5). Each condition was carried out on 3 biological replicates (*n* = 3). Significant changes for only siRNA comparisons are starred: *P ≤ 0.05; **P ≤ 0.01; ***P ≤ 0.001 (*T*-test) and all *P*-values for this figure including comparisons of 24 and 48 h hypoxia exposure with normoxia are in Table S4.

Glycosyltransferases expression in all cell lines was in general dysregulated in all hypoxia conditions (Figure 5). Overlaying multiple regulatory tracks with the human genome (using the UCSC genome browser, http://genome-euro.ucsc.edu/), we were able to identify transcription factors (GATA2 and GATA3) that potentially regulate MGAT5 and ST3GAL4 in our ovarian cancer cell lines. These tracks included transcription factor CHIP-seq clusters from the ENCODE project [Encyclopedia of DNA Elements, (35)], CpG island information, DNAase hypersensitivity information (also from ENCODE) and TFBS (Transcription Factor Binding Site) conservation information across human mouse and rat. This showed that both of these transcription factors have regulatory target sites in the promoter regions of both MGAT5 and ST3GAL4 confirmed by CHIP-seq, in likely promoter regions confirmed by DNAase hypersensitivity, the presence of a CpG island, and cross-species conservation. An siRNA targeted inhibitory approach was undertaken to confirm this. GATA2 expression was assessed by Western blotting and qRT-PCR in the ovarian cancer isogenic cell line pair, A2780 and A2780cis, and GATA3 expression in the isogenic PEO1 and PEO4 pair of cell lines, cultured in 0.5% hypoxia. This 0.5% percentage of hypoxia was chosen as culturing in this exposure showed the most significant alterations in glycosylation and migration. Results show that the expression levels of GATA TFs and the glycosyltransferases were decreased after 24 h hypoxia (0.5%) and increased after 48 h hypoxia (0.5%) exposure in all cell lines at the protein expression levels and mostly at the mRNA expression level (Figure 6). When GATA TFs mRNA expression levels were knocked down using siRNA, the GATAs and glycosyltransferase levels were mostly significantly decreased at 48 h hypoxia (0.5%) exposure, but not at 24 h 0.5% exposure (Figure 6, Figure S5, Table S4). This observation, while present at the protein level (Figure 6B) was more pronounced at the gene expression level (Figure 6A, Table S4).

## Discussion

From the literature, we know that epigenetics, glycosylation and hypoxia, play key roles in cancer progression, cancer cell survival and treatment response (36). However, little is known as to how all three may potentially be integrated in this process.

### DNA Methylation Alters in Hypoxia in a Time-Dependent Manner, and May Be Related to the Oxygen Dependant Enzyme TET

Firstly, the impact that hypoxia exposure (0.5–2% O_2_) might have in altering DNA methylation was assessed. Results demonstrated that following differential exposure to hypoxia for 24 and 48 h, significant changes in global DNA methylation were observed in all six cell lines, in comparison to cells cultured in normoxic (21% O_2_) conditions. Specifically, DNA methylation increased significantly after 24 h, but decreased significantly after 48 h (*p* ≤ 0.05). This was observed predominantly for the 0.5 and 1% O_2_ exposure. Observed changes in DNA methylation following 2% O_2_ exposure were small and not significant (Figure 1). While the paradigm of hypoxia inducing global DNA demethylation with site specific hypermethylation, is a widely accepted finding (37), the shift from increased to decreased DNA methylation between the 24 and 48 h hypoxic exposure times has not been observed previously. This may possibly be explained by the action of the oxygen dependant enzyme Ten Eleven Translocation (TET). TET demethylates DNA through the oxidation of 5-methylcytosine (5-meC), in a site-specific manner and has previously been shown to have reduced activity after 24 h of 0.5% hypoxia exposure (37).

### Increases in Larger More Branched and Sialylated Glycans on Secreted Glycoproteins From Cancer Cells in Hypoxia Aids to Their Metastatic Capabilities

In relation to the *N*-glycan profiles, significant changes in glycosylation were observed in 0.5–2% hypoxia, namely **a decrease** in core fucosylated and high mannosylated glycans, and **an increase** in outer arm fucosylation, polylactoseamine extensions and sialylation in all cell lines. The only exception was for the ovarian cancer cell line PEO1 (Figure 2). The observation for the PEO1 cells may reflect the fact that they have a longer doubling time than the other cell lines used (38, 39), which could impact on *N*-glycan signatures. This increase in the larger triantennary sialylated structures has been previously been linked with the formation of selectin binding structures, promoting extravasation of circulating tumor cells, invasion and adhesion to lymph nodes, thereby supporting metastasis (40). Interestingly, we found substantial levels of ketodeoxynonulosonic acid (KDN) in all our cell lines (Table S2). KDN has been found in various cancer tissues including ovarian cancer, and the ratio of KDN levels to other sialic acid types (Neu5Ac and Neu5Gc), has been found to be predictive of the metastatic potential of cells. Importantly, hypoxia regulates the expression of KDN processing enzymes, resulting in increased update of KDN precursors from the environment (41). Interestingly, this form of sialic acid is normally found in lower vertebrates and on the cell wall polysaccharides of pathogenic bacteria (42). This observation may indicate the presence of these bacteria in cancer cells and cancer tissues (43–45). In the present study, there were no significant changes in KDN (GP29) in hypoxia conditions, with the exception of the PEO4 cells where increased expression of KDN was observed at 24 h in 1% hypoxia (Figure 2).

### Increases in EMT and Migration of Cancer Cells in Hypoxia Increases Their Aggressiveness and Invasive Potential

The metastatic capability of all 6 cell lines was assessed using a combination of the migration assay (Oris cell assay) and determining the relative expression of proteins known to be involved in cell adhesion and cell motility. All cell lines grown in 0.5% hypoxia demonstrated a significant increase in migration compared to cells grown in normoxia (21% O_2_) (Figure 3). This increased migration was maintained in 1% hypoxia and 2% hypoxia for the A2780 and MDA-MB-231 cell lines, respectively. Glycosylation, EMT and migration of PEO1 and MDA-MB-436 in a hypoxic environment, was the least affected when compared to the other cell lines (Figures 2, 3). In general, longer hypoxia exposure (48 h) and lower O_2_ percentages (0.5% O_2_) was associated with more significant increases in EMT (expression of E-cadherin, vimentin and N-cadherin) and cell migration in all cell lines. It is well-documented that hypoxia induces migration and increases tumor cell aggressiveness reflecting the many pathways that are HIF-regulated. The main contributor in the literature appears to be HIF1-α (46), which has shown to be increased in all hypoxia exposed cell lines (Figure 4). Another mechanism which is involved in hypoxia induced migration is the unfolded protein response (UPR) mechanism (47). The UPR occurs in the endoplasmic reticulum (ER), when misfolded proteins build up due to high metabolic rates, limited supplies of glucose, and extreme decreases in O_2_ availability (48). The ER is also the start site of protein glycosylation. Hypoxia induced UPR is also implicated in the induction of EMT in many cancers, including breast and ovarian (49–51). The changes observed in EMT, migration and glycosylation of cell secreted glycoproteins following hypoxic exposure, particularly at the 0.5 and 1% O_2_ levels may enhance the invasive potential of these cells in highly hypoxic tumors such as ovarian and Triple Negative Breast Cancers (TNBC).

### Senescence and Autophagy Alterations in Hypoxia Promote the Cell Proliferation and Metastasis, Apoptosis Increases Only in Chemosensitive Cell Lines

Alterations in markers which are well-established with other cellular events such as apoptosis and senescence were examined. Interestingly, PARP cleavage, indicative of cellular apoptosis, showed an increase in chemosensitive cells (A2780 and PEO1), but a decrease in chemoresistant cell lines (A2780cis, PEO4 and MDA-MB-436) (Figure 4). LC3 autophagy marker was increased in all cells lines in hypoxia conditions, although significantly only in A2780cis, PEO1, and PEO4, and this marker was not expressed in MDA-MB-231 in the given conditions (Figure 4). Tumor cells use autophagy to support self-proliferation and metastasis; leading to poor disease prognosis (52), which indicates that hypoxia aids to these tumor features. It is of note that while p21 was decreased in the PEO4 cell line with increasing hypoxia, there was a significant increase in the protein levels of LC3 possibly reflecting the innate chemoresistance of this cell line and the need to maintain viability.

### Hypoxia May Impact GATAs Expression Through Regulation of DNA Methylation Levels and GATAs Then Increase Expression of Highly Branched and Sialylated Glycans Through Increases in Particular Glycosyltransferases

To shed more light on the origin of glycan alterations as well as the changes in cell migration and EMT in hypoxia, especially at the lower oxygen levels (0.5%), the expression of the glycosylation enzymes, previously found dysregulated in the ovarian cancer cell line OVCAR3 under demethylation conditions, was investigated (53) (Figure 5). This list of enzymes included enzymes involved in branching and sialylation of *N*-linked glycans, specifically *MGAT5* and *ST3GAL4*. Potential TFs, *GATA1-3* for these glycosyltransferase enzymes, had previously been identified *in silico*. An siRNA targeted inhibitory approach was undertaken to see how the expression of these glycosyltransferases was affected. Results demonstrated that the protein expression levels of the GATAs and the glycosyltransferase levels were decreased at 48 h 0.5% hypoxia exposure, but not at 24 h (Figure 6, Figure S5).

These increases or decreases were significant mostly at the mRNA levels, some results were also evident at the protein level (*p* ≤ 0.05) (Figure 6, Table S4). Interestingly, there was a decrease in the expression levels of both GATA2 and 3 and MGAT5 and ST3GAL4 glycosyltransferases after 24 h of hypoxia exposure with increased expression after 48 h, both at the mRNA and protein level (Figure 6). This was in contrast to the DNA methylation levels (Figure 1), where DNA methylation was increased after 24 h of hypoxia exposure and decreased after 48 h (Figure 1). GATA3 interacts and stabilizes the HIF protein and increases tumor cell migration and invasiveness under hypoxic conditions (10). GATA2 has not as yet been linked with hypoxia. However, it is known that DNA methylation is important for GATA2 transcriptional regulation (54). Therefore, we hypothesize that hypoxia impacts on the expression of GATAs, through regulation of global DNA methylation levels. Increased expression of GATA TFs then increases the expression of MGAT5 and ST3GAL4 glycosyltransferases, leading to increases in levels of highly branched and sialylated glycans and their expression aids to increase/enhance the migration of the ovarian and breast cancer cell lines in hypoxic conditions. Further studies are warranted to test that hypothesis to focus on the proposed GATA2/3 and ST3GAL4/MGAT5 mechanism, in relation to the specific alterations observed in secreted *N*-linked glycomes.

## Conclusion

Global hypermethylation was observed at 24 h and global hypomethylation at 48 h in all cell lines exposed to 0.5–2% hypoxia. Concomitantly, glycosylation on secreted cell glycoproteins changed to highly branched and sialylated glycan structures. These same structures are known to be involved in increased aggressiveness and increased metastatic capabilities (41). In hypoxia, these ovarian and breast cancer cells also demonstrated increased expression of EMT markers and an increased migratory potential. It is well-documented that DNA methylation is an important regulator of the GATA2 and GATA3 transcription factors (TFs). When GATA TFs levels decreased after siRNA knockdown, the levels of MGAT5 and ST3GAL4, directly involved in *N-*glycan branching and sialylation, were also decreased at 48 h hypoxia (0.5%) exposure. Potentially, hypoxia may act as a driver of GATA regulation through its altered DNA methylation. While it has be shown that GATA3 is required for the stabilization of a hypoxic microenvironment through binding with HIF1-α, this current study shows the possible role of GATA 2 and GATA3 in altering the glycosylation of tumor glycoproteins, thereby potentially contributing to cell invasiveness. In a recent study, a GATA3-specific DNAzyme (hgd40) was used to inhibit GATA3, which resulted in successfully protecting mice from chronic inflammation caused by ulcerative colitis (55). A similar study has yet to be performed in cancer. In summary, this is the first *in vitro* study to investigate DNA methylation, hypoxia and *N*-glycan profiling together in the *in vitro* setting, and highlights the complexity of these key integrated tumor microenvironmental features.

## Data Availability Statement

The original contributions presented in the study are included in the article/Supplementary Material, further inquiries can be directed to the corresponding author.

## Author Contributions

PR, AM, and RS conceived and initiated this study. GG, EL, CH, JC-F, SP, RO'F, and RS carried out the experiments. GG, EL, CH, RO'F, and RS generated and interpreted the results. EL, RP, SM, and RS conducted the statistical analyses. GG, EL, RP, PR, AM, and RS drew the conclusions. GG and RS with significant input from AM wrote the manuscript which was reviewed by all co-authors.

## Conflict of Interest

The authors declare that the research was conducted in the absence of any commercial or financial relationships that could be construed as a potential conflict of interest.

## Supplementary Material

Figure S1 GATA3 concentration optimisation.

Figure S2 Western blot images of the EMT markers, E-cadherin, N-cadherin and Vimentin, in hypoxia treated cells (*n* = 3, shown are representative blots). Images show normoxia (21% O_2_), 0.5, 1, and 2% after 24 and 48 h exposure.

Figure S3 Histogram profiles representing the percentage phases of the cell cycle, G1, S, G2, before and in hypoxia. All experiments represent three biological experiments are *n* = 3.

Figure S4 Hypoxia alters senescence, apoptosis and autophagy specifically. Representative Western blots of the (i) senescence associated proteins (p16, Rb, p21, (ii) autophagy associated LC3 and (iii) the apoptosis marker PARP of 4 ovarian cancer cell lines and 2 TNBC cell lines in differential hypoxia compared to normoxia control cultures (21% O_2_). Hypoxia induction is confirmed by appreciation of the hypoxia marker HIF1α. All results represent three biological experiments (*n* = 3).

Figure S5 SiGATA2/3 knockdown alters the levels of MGAT5 and ST3GAL4. Western blot analysis of siGATA2/3 knockdown in 4 ovarian cancer cell lines. GATA2 was knocked down in A2780 and A2780cis and GATA3 was knocked down in PEO1 and PEO4 in hypoxia exposed cells compared to controls. All results represent three biological experiments (*n* = 3).

Table S1 List of all glycans from all 6 cell lines and how they were pooled into features.

Table S2 Detailed assignments of *N*-glycans from secreted glycoproteins of all 6 cell lines using UPLC and exoglycosidase digestions.

Table S3 Mass spectrometry data in negative mode of secreted *N*-glycome from cell glycoproteins.

Table S4 *P*-values for siGATA knock down experiments (Figure 6).
