# Supplementary figures and images for: Hypoxia Alters Epigenetic and N-Glycosylation Profiles of Ovarian and Breast Cancer Cell Lines in-vitro
Source: Front Oncol. 2020 Jul 29;10:1218. doi: 10.3389/fonc.2020.01218 (PMC7405916; doi:10.3389/fonc.2020.01218)

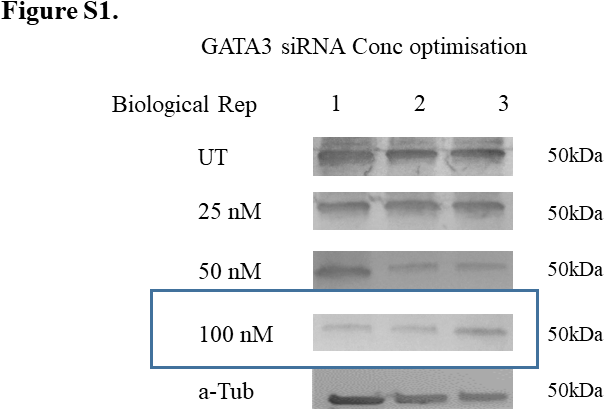

Supplement: Figure S1 — GATA3 concentration optimisation. [file Image_1.TIF]

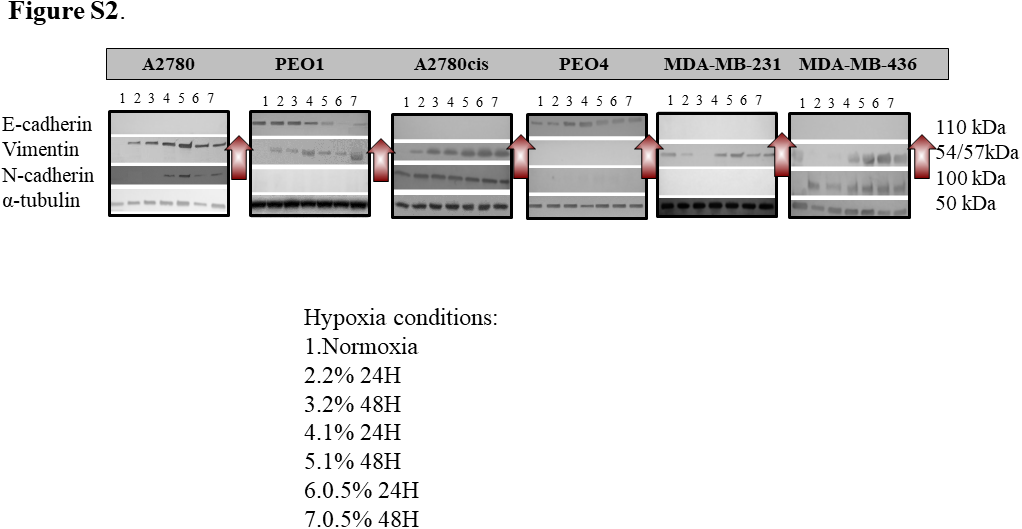

Supplement: Figure S2 — Western blot images of the EMT markers, E-cadherin, N-cadherin and Vimentin, in hypoxia treated cells (n = 3, shown are representative blots). Images show normoxia (21% O2), 0.5, 1, and 2% after 24 and 48 h exposure. [file Image_2.TIF]

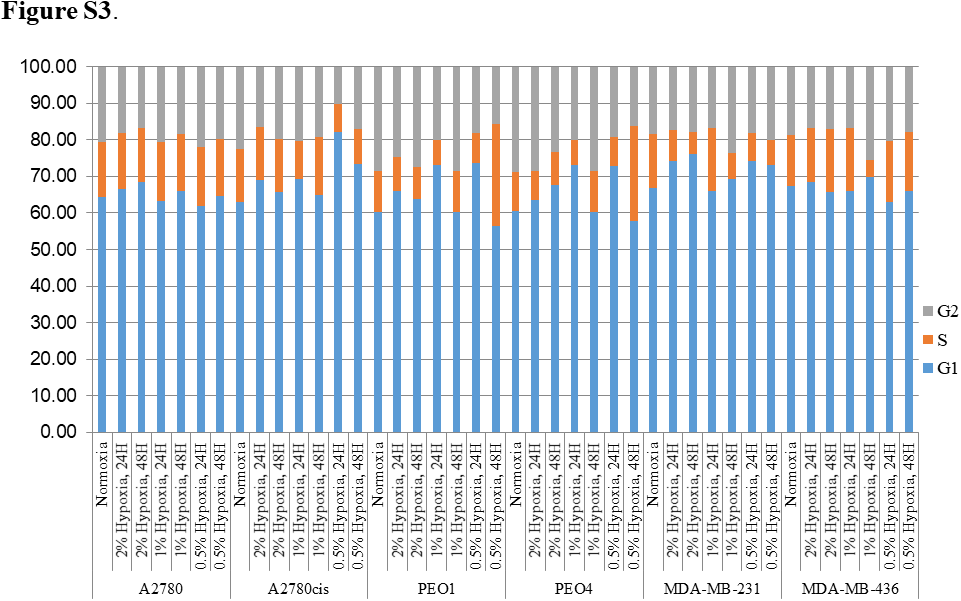

Supplement: Figure S3 — Histogram profiles representing the percentage phases of the cell cycle, G1, S, G2, before and in hypoxia. All experiments represent three biological experiments are n = 3. [file Image_3.TIF]

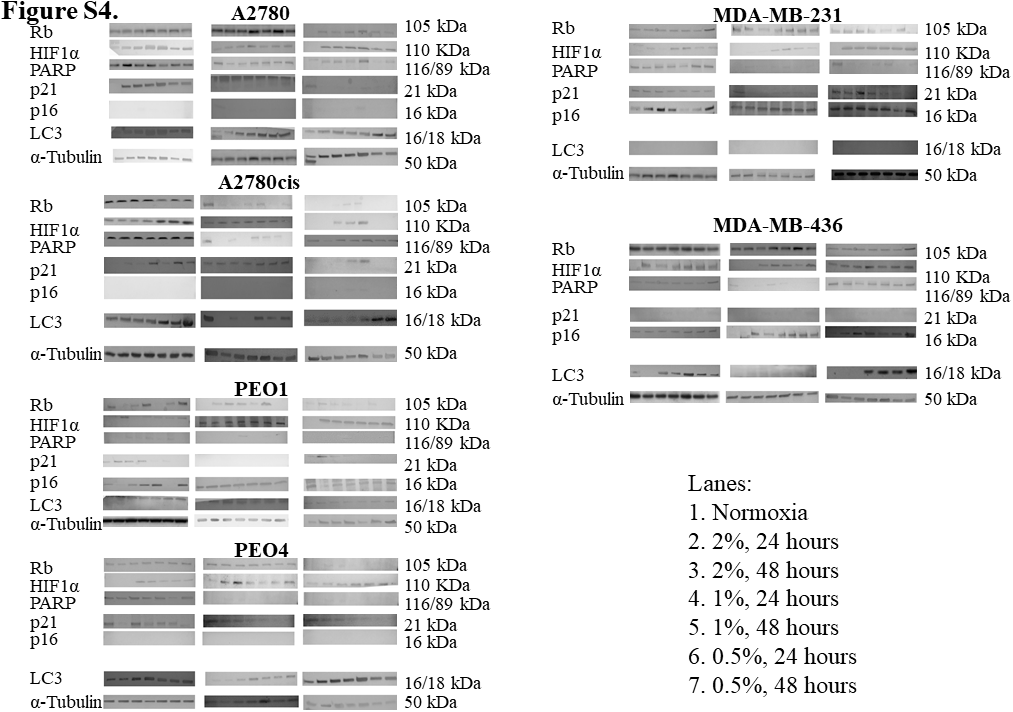

Supplement: Figure S4 — Hypoxia alters senescence, apoptosis and autophagy specifically. Representative Western blots of the (i) senescence associated proteins (p16, Rb, p21, (ii) autophagy associated LC3 and (iii) the apoptosis marker PARP of 4 ovarian cancer cell lines and 2 TNBC cell lines in differential hypoxia compared to normoxia control cultures (21% O2). Hypoxia induction is confirmed by appreciation of the hypoxia marker HIF1α. All results represent three biological experiments (n = 3). [file Image_4.TIF]

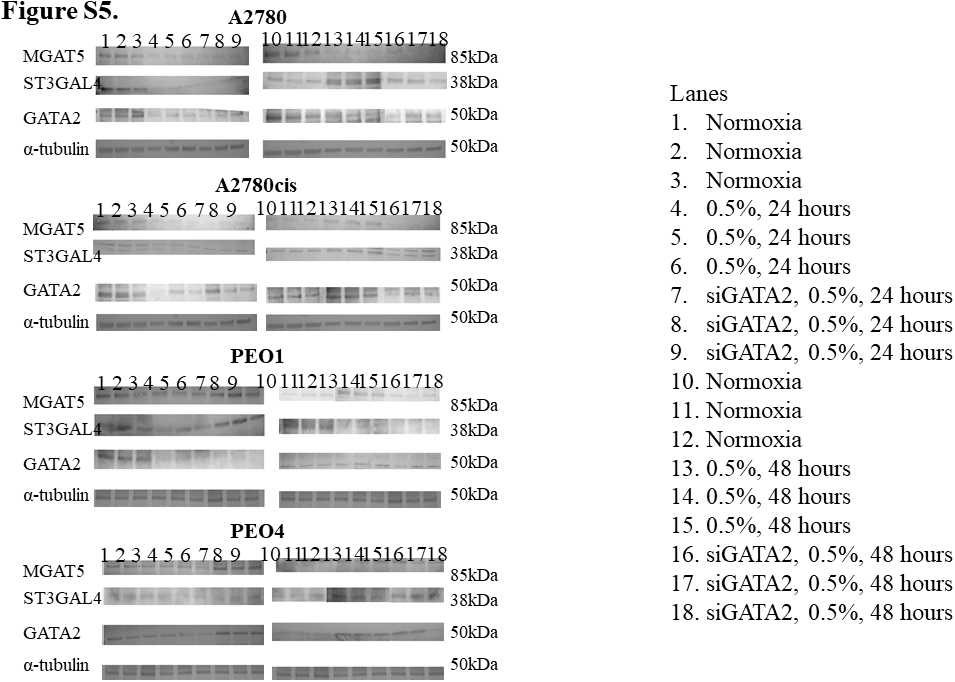

Supplement: Figure S5 — SiGATA2/3 knockdown alters the levels of MGAT5 and ST3GAL4. Western blot analysis of siGATA2/3 knockdown in 4 ovarian cancer cell lines. GATA2 was knocked down in A2780 and A2780cis and GATA3 was knocked down in PEO1 and PEO4 in hypoxia exposed cells compared to controls. All results represent three biological experiments (n = 3). [file Image_5.TIF]
